# Use of cardiopulmonary exercise testing to assess early ventilatory changes related to occupational particulate matter

**DOI:** 10.1590/1414-431X20186486

**Published:** 2018-03-26

**Authors:** T.P. Chao, E.F. Sperandio, T.L.V.P. Ostolin, V.R. Almeida, M. Romiti, A.R.T. Gagliardi, R.L. Arantes, V.Z. Dourado

**Affiliations:** 1 Laboratório de Epidemiologia e Movimento Humano, Departamento de Ciências do Movimento Humano, Universidade Federal de São Paulo, Santos, SP, Brasil; 2 Instituto de Medicina Cardiovascular Angiocorpore, Santos, SP, Brasil

**Keywords:** Occupational exposure to particulate matter, Cardiopulmonary exercise testing, Detection, Spirometry, Ventilatory impairment

## Abstract

Spirometry has been used as the main strategy for assessing ventilatory changes related to occupational exposure to particulate matter (OEPM). However, in some cases, as one of its limitations, it may not be sensitive enough to show abnormalities before extensive damage, as seen in restrictive lung diseases. Therefore, we hypothesized that cardiopulmonary exercise testing (CPET) may be better than spirometry to detect early ventilatory impairment caused by OEPM. We selected 135 male workers with at least one year of exposure. After collection of self-reported socioeconomic status, educational level, and cardiovascular risk data, participants underwent spirometry, CPET, body composition assessment (bioelectrical impedance), and triaxial accelerometry (for level of physical activity in daily life). CPET was performed using a ramp protocol on a treadmill. Metabolic, cardiovascular, ventilatory, and submaximal relationships were measured. We compared 52 exposed to 83 non-exposed workers. Multiple linear regressions were developed using spirometry and CPET variables as outcomes and OEPM as the main predictor, and adjusted by the main covariates. Our results showed that OEPM was associated with significant reductions in peak minute ventilation, peak tidal volume, and breathing reserve index. Exposed participants presented shallower slope of ΔVT/ΔlnV̇E (breathing pattern), i.e., increased tachypneic breathing pattern. The OEPM explained 7.4% of the ΔVT/ΔlnV̇E variability. We found no significant influence of spirometric indices after multiple linear regressions. We conclude that CPET might be a more sensitive feature of assessing early pulmonary impairment related to OEPM. Our cross-sectional results suggested that CPET is a promising tool for the screening of asymptomatic male workers.

## Introduction

Occupational exposure to particulate matter (OEPM) is responsible for the increase in mortality and morbidity rates in workers by cardiorespiratory diseases (1–3). Spirometry has been used as the first-choice instrument for the evaluation of pulmonary alterations in individuals vulnerable to OEPM (4,5). Currently, the instrument is indicated for diagnosing the risk of damage, identifying lung disease, monitoring subjects exposed to harmful particulate matter, as well as to evaluate therapeutic interventions (6). However, in some cases, as one of its limitations, it might not be sensitive enough to show abnormalities before extensive damage as seen in restrictive lung diseases (7).

The evaluation of cardiorespiratory fitness with cardiopulmonary exercise testing (CPET) is used for the diagnosis of exercise intolerance as well as prognosis of patients with chronic diseases (8). Because of the complexity of using exercise testing, imaging information regarding cardiac structure and function as well as ventilatory gas exchange measurements are used to detect small changes that reflect the functional capacity of the cardiovascular, respiratory, and musculoskeletal systems. These measurements respond to different metabolic demands in health and disease conditions (9). Therefore, compared to spirometry, CPET could be a more sensible respiratory test to detect early ventilatory impairment caused by OEPM.

CPET has been explored in the literature regarding its prognostic and diagnostic value in cardiorespiratory diseases, as well as in cardiac and pulmonary rehabilitation. However, there are few studies in the literature focusing on the potential of CPET for screening early pulmonary alterations caused by OEPM. It has been shown that CPET is an appropriate tool to assess heterogeneous abnormalities related to respiratory mechanics and pulmonary gas exchange that are not detected early by traditional resting pulmonary function tests, e.g., spirometry. CPET can also explain the incipient symptoms of dyspnea and exercise intolerance in any given individual. Moreover, CPET may identify the effects of skeletal muscle dysfunction differentiating between physical inactivity causes from impaired cardio-circulatory function. The absence of abnormal physiologic responses to CPET in a symptomatic subject requires careful evaluation (10). Thus, the aim of this study was to compare spirometry and CPET to detect ventilatory alterations in workers exposed to OEPM. The hypothesis is that CPET might be more efficient than spirometry to assess incipient ventilatory abnormalities.

## Material and Methods

### Study design

We conducted a cross-sectional study involving male workers. Participants were selected from the beginning of the epidemiology and human movement study (EPIMOV Study). Briefly, the EPIMOV Study is a population-based cohort study, which the main purpose is to investigate the associations of physical inactivity and sedentary behaviors with the occurrence of hypokinetic diseases. The Ethics and Research Committee on Human Beings of the Universidade Federal de São Paulo (UNIFESP) approved the study (#186.796). All participants signed a consent form.

Demographic characteristics (age, gender, socioeconomic status, and educational level), self-reported medical history, anthropometrics, respiratory questionnaire (11), cardiovascular risk, spirometry, CPET, body composition (bioelectrical impedance), and level of physical activity in daily life (PADL) were collected from participants.

We included male workers older than 18 years with normal pulmonary function (n=153) and excluded those who had musculoskeletal problems and lung and heart diseases previously diagnosed or identified during evaluation, previous thoracic surgery, previous history of mechanical ventilation, were using assistive gait devices, or had others problems identified in the electrocardiogram and issues that the researchers judged impeding for performing physical exercises safely.

Our exposure group (EG) and control group (CG) were defined according to a validated occupational respiratory questionnaire (i.e., ATS-DLD78), which was applied before the spirometric test. Those who answered “yes” to OEPM were included in the EG and who answered “no” were included in the CG.

The EG group was composed of individuals who reported exposure to many types of particulate matter during work, especially from port activities in Santos, SP, Brazil.

### Anthropometry

Body mass to the nearest 0.1 kg and height to the nearest 0.1 cm were measured by a digital scale with a stadiometer (Toledo®, Brazil) according to standard recommendations previously described (12). The body mass index was then calculated (kg/m^2^).

### Respiratory assessment

We applied a respiratory questionnaire based on a previously validated questionnaire developed by the American Thoracic Society (ATS) (11) to investigate pulmonary impairments and OEPM, which comprised: “Have you had any lung disease?”, “Do you have or have had asthma?”, “Have you ever underwent any thoracic surgery?”, “Have you had to breathe through invasive mechanical ventilation?”, “Have you ever worked in environments with high dust, smoke or other suspended chemicals for at least 1 year?”

Spirometry measurements were performed using a periodically calibrated spirometer (Quark PFT, Italy), according to the criteria established by the ATS (6). Forced vital capacity (FVC), forced expiratory volume in 1 s (FEV_1_), and FEV_1_/FVC ratio were analyzed. Obstructive lung disease was identified by FEV_1_/FVC <0.7. Spirometric restrictive patterns were considered for FVC values <80% of the predicted values (13) with FEV_1_/FVC >0.7.

### Cardiopulmonary exercise testing

CPET was performed on a treadmill ramp protocol (ATL, Ibramed, Brazil) in which the speed and inclination increase were individualized according to the predicted peak oxygen uptake (peak V̇O_2_). We designed CPETs to be completed in 10 min on average (range 8–12 min). Metabolic, cardiovascular, and ventilatory responses were measured breath by breath through a gas analyzer (Quark PFT, Cosmed, Italy). The necessary air, gas mixture, and syringe calibrations were performed according to the manufacturer's recommendations. Pulmonary oxygen uptake (V̇O_2_), carbon dioxide production (V̇CO_2_), and minute ventilation (V̇E) were measured throughout CPET and data were filtered through an arithmetic average every 15 s for further analysis. Heart rate (HR) was monitored throughout the test through a 12-lead electrocardiography (C12x, Cosmed). The anaerobic threshold (AT) was estimated by the v-slope method. The breathing reserve was calculated by subtracting the peak V̇E of maximum voluntary ventilation (MVV) estimated by age and gender of the participants (14). Subsequently, the breathing reserve index (BRI) was calculated by the following equation [(peak V̇E/MVV) × 100] and reported as percentage, i.e., the higher the value, the lower the breathing reserve. Maximal exercise was determined in case of at least one of the following physiological indices: peak HR>85% of predicted for age (220 – age), peak V̇CO_2_/V̇O_2_ >1.0, or oxygen uptake plateau (i.e., V̇O_2_ maximum). Exercise intolerance was considered when peak V̇O_2_ was below 83% of the predicted values (15).

Additionally, the following submaximal relationships were determined throughout CPET: ΔHR/ΔV̇O_2_, ΔV̇E/ΔV̇CO_2_, and ΔVT/ΔlnV̇E. These relationships were obtained by linear regressions as previously described (14) and represent cardiovascular and ventilatory efficiency, and breathing pattern throughout the exercise, respectively.

We applied the natural logarithm in ΔVT/ΔlnV̇E as a strategy to linearize the relationship between VT and VE since the increase of tidal volume is not linear during the maximal incremental test in relation to ventilation. This strategy allowed us to calculate the slope of the relationship in a simple linear regression.

### Body composition

Body composition was determined by bioelectrical impedance (310E Biodynamics, USA). The resistance and reactance were obtained according to the standards and protocols developed by Kyle et al. (16). The lean body mass (LBM) and fat mass were calculated using the group-specific equations for healthy individuals (17).

### Physical activity in daily life

The triaxial accelerometer (ActiGraph, MTI, USA) (17,18) was attached to the waist by an elastic band above the dominant hip to evaluate the amount and intensity of PADL. The evaluations were performed over the course of 7 days. The validation of the data was based on the use of the device for at least 4 valid days with at least 720 min of monitoring. The time spent in sedentary, light, moderate, vigorous, and very vigorous physical activity was recorded and the number of steps/day was measured. We considered the minimal recommendations of PADL of 30 min of moderate to vigorous physical activity for at least 5 days per week (19). Those who had not performed this level of PADL were considered physically inactive.

### Statistical analysis

Statistical analysis was performed using SPSS software version 23 (SPSS Inc., USA), with a significance of P<0.05. The normality of the sample was verified using the Kolmogorov-Smirnov test. Categorical variables are reported as frequency and percentage and we subsequently applied the chi-square test to verify the differences between groups. The association between categorical variables and OEPM was evaluated through 2×2 tables. Odds ratios (OR) and 95% confidence intervals (95%CI) were calculated. Continuous variables are reported as means±SD, and median and interquartile range (25–75%). The Student's *t*-test or Mann-Whitney test were applied in the continuous variables to determine if there were significant differences between groups.

Multiple linear regression models were developed considering the spirometric indices and responses to CPET as primary outcomes, and OEPM as the main predictor. After bivariate analyses, we adjusted the multivariate models by the main confounders: age, gender, height, LBM, sedentary physical activity level (%), socioeconomic status, education level, hypertension, diabetes mellitus, dyslipidemia, obesity, and smoking. These variables have been identified as correlated to pulmonary function and were included to check their influence in the multivariate analysis.

The sample size calculation took into account the number of clinically relevant predictors. We considered a total correlation coefficient of r=0.80 and a coefficient of determination of R^2^=0.64. With alpha error of 0.05 and beta error of 0.80, we achieved a sample of 10 observations for each variable included in the model. Considering that the models were adjusted for 12 variables, the sample was sufficient to answer our research question.

## Results

Fifty-two participants reported “yes” to OEPM (”Have you ever worked in environments with high dust, smoke or other suspended chemicals for at least 1 year?”) and were allocated in EG. The remaining 83 subjects composed the CG.

EG presented older age, larger amount of lean body mass (kg), and lower educational level (Table 1). There was a significant association between higher educational level and OEPM (OR=0.290: 95%CI=0.100-0.842). EG reported many types of occupations with exposure to a large amount of particulate matter, especially materials from Santos port activities.

**Table 1.** General characteristics of the participants according to occupational exposure to particulate matter.

Variables	CG (n=83)	EG (n=52)
Age (years)*	37±11	41±12
Height (m)*	1.74±0.07	1.70±0.07
Weight (kg)	86.5±16.3	81.6±13.4
Body mass Index (kg/m^2^)	28±5	28±4
Body composition		
Body fat (%)	23±7	24±5
Body fat (kg)	21±10	20±7
Lean body mass (%)	77 (70-84)	74 (69-79)
Lean body mass (kg)*	64 (57-74)	61 (58-64)
Socio-economic level		
Low class	19 (22.9%)	25 (48.1%)
Medium class	35 (42.2%)	10 (19.2%)
High class	16 (19.3%)	12 (23.1%)
Educational level (incomplete high school)*	6 (7.2%)	11 (21.2%)
Cardiovascular risk		
Hypertension	6 (7.2%)	8 (15.4%)
Diabetes mellitus	3 (3.6%)	4 (7.7%)
Dyslipidemia	13 (15.7%)	13 (25.0%)
Obesity	23 (27.7%)	13 (25.0%)
Smoking	6 (7.2%)	8 (15.4%)
Physical inactivity	14 (16.9%)	4 (7.7%)

Categorical data are reported as frequency (%). Variables with normal distribution are reported as means±SD, with non-normal distribution as median (interquartile range). CG: control group (not exposed); EG: exposed group. *P<0.05 intergroup comparison (chi-square test for categorical data and Student's *t*-test or Mann-Whitney test for continuous variables).

Although the absolute values of FEV1 and FVC were significantly different, we did not find spirometric differences in FEV_1_ (%) and FVC (%) between groups (Table 2).

**Table 2.** Comparison of spirometry data among participants exposed and not exposed to occupational particulates.

Variables	CG (n=83)	EG (n=52)
FVC (L)*	4.81±0.79	4.49±0.78
FVC (%)	97±11	95±10
FEV_1_ (L)*	3.86±0.68	3.61±0.71
FEV_1_ (%)	95±12	93±12
FEV_1_/FVC	0.81±0.05	0.80±0.06
PEF (L/s)	9.94±1.76	9.40±1.96

Data are reported as means±SD. CG: control group (not exposed); EG: exposed group. FVC: forced vital capacity; FEV_1_: forced expiratory volume in 1 s; PEF: peak expiratory flow.

* P<0.05 intergroup comparison (Student's *t*-test or Mann-Whitney test).

Regarding PADL, participants of the EG showed a higher weekly amount of sedentary (77±8 *vs* 74±8%) and very light physical activity (14±5 *vs* 12±4%).

The influence of moderate-to-vigorous physical activity was analyzed as a categorical variable instead of as a continuous variable (i.e., physical inactivity) in multivariate analysis. We found no differences between groups.

Ten participants (19.2%) in the EG and 8 (9.6%) in the CG presented peak V̇O_2_ indicating exercise intolerance (P=0.09). EG presented worse physiological responses to CPET. There were significant differences for peak V̇O_2_, V̇E, VT, BRI, and end-expiratory pressure of O_2_. Regarding submaximal responses, the EG presented worse ΔHR/ΔV̇O_2_, ΔVT/ΔlnV̇E, and absolute values of AT. Moreover, the AT as percentage of predicted peak V̇O_2_ tended to be lower in the EG (Table 3).

**Table 3.** Maximal and submaximal physiological responses obtained during cardiopulmonary exercise test in stratified participants according to occupational exposure to particulate matter.

Variables	CG (n=83)	EG (n=52)
Test duration (min)	9.34±1.28	9.15±1.45
Maximum metabolic response		
V̇O_2_ (mL/min)*	3197 (2666-3631)	2816 (2159-3454)
V̇O_2_ (mL·kg^-1^·min^-1^)*	38.2±9.3	35.4±9.6
V̇O_2_ (% pred)*	101±15	95±17
Metabolic equivalent	11±3	10±3
V̇CO_2_/V̇O_2_ *	1.22±0.10	1.17±0.10
PetCO_2_ (mmHg)	42.8±3.5	42.9±4.5
PetO_2_ (mmHg)*	111±3	109±4
Maximum ventilatory response		
V̇E (L/min)*	107±21	91±25
BRI (%)*	67±14	61±13
VT (L)*	2.58±0.48	2.26±0.42
f (rpm)	41±7	40±7
V̇E/V̇O_2_	33.6±3.9	32.3±4.8
V̇E/V̇CO_2_	27.9±3.1	28.3±4.1
Maximum cardiovascular response		
HR (%pred.)	94±8	95±7
V̇O_2_/HR (mL·min^-1^·bpm^-1^)	18.5±3.9	17.0±4.7
Systolic blood pressure (mmHg)	185 (170-200)	180 (170-190)
Submaximal relationship		
AT (mL/min)*	2058 (1675-2498)	1851 (1463-2150)
AT (% of VO_2_peak)	66±12	66±9
AT (%V̇O_2_ pred. peak)	66 (52-78)	59 (52-73)
ΔHR/ΔV̇O_2_ (bpm·L^-1^·min^-1^)	35.5±9.0	38.0±9.2
ΔV̇E /ΔV̇CO_2_	24.4±3.1	24.5±3.6
ΔVT/ΔlnV̇E*	0.94±0.27	0.82±0.18

Data are reported as means±SD or median and interquartile range. CG: control group (not exposed); EG: exposed group; V̇E: minute ventilation; V̇O_2_: oxygen uptake; V̇CO_2_: carbon dioxide production; BRI: breathing reserve index; VT: tidal volume; f: respiratory rate; HR: heart rate; PetCO_2_: end-expiratory pressure of CO_2_; PetoO_2_: end-expiratory pressure of O_2_; AT: anaerobic threshold, ΔHR/ΔV̇O_2_: cardiac efficiency; ΔV̇E/ΔV̇CO_2_: ventilatory efficiency; ΔVT/ΔlnV̇E: breathing pattern.

* P<0.05 intergroup comparison (Student's *t*-test or Mann-Whitney test).

In the multiple regressions, exposure to OEPM was not identified as an independent predictor of spirometric indices. The differences found in absolute values of FEV_1_ and FVC were attributed to difference in age and height. Regarding CPET, OEPM was identified as an independent predictor for peak V̇E, BRI, peak VT, and ΔVT/ΔlnV̇E (Table 4). Therefore, OEPM was not a determinant for other measures of CPET.

**Table 4.** Significant associations between occupational exposure to particulate matter and responses obtained in the cardiopulmonary exercise testing.

Outcomes	Occupational exposure to particulate matter			
	B (SE)	P	ΔR^2^	Total R^2^
V̇E peak (L/min)	−8.451 (0.162)	0.028	0.043	0.407
BRI (%)	−7.880 (3.009)	0.010	0.063	0.114
VT peak (L)	−0.251 (0.088)	0.006	0.068	0.192
ΔVT/ΔlnV̇E	−0.139 (0.051)	0.007	0.074	0.074

Linear multiple regression models adjusted for age, gender, height, lean body mass, sedentary physical activity, socioeconomic status, educational level, hypertension, diabetes mellitus, dyslipidemia, obesity, and smoking. V̇E: minute ventilation; BRI: breathing reserve index; VT: tidal volume; ΔVT/ΔlnV̇E: breathing pattern; SE: standard error.

## Discussion

The main findings of the present study showed that male workers exposed to OEPM for at least one year exhibited ventilatory alterations during exercise, such as shallower ΔVT/ΔlnV̇E and peak V̇E, even with normal pulmonary function at rest. CPET has proven to be useful in identifying these early ventilatory changes related to OEPM.

Both CG and EG presented normal exercise responses, with no significant differences in the diagnosis of exercise intolerance (P=0.09). We found shallower ΔVT/ΔlnV̇E in the EG and this was determined solely by OEPM (R^2^=0.074). Moreover, we observed that OEPM was associated with the reduction of 8.4 L/min in peak V̇E and 0.25 L in peak VT in CPET. Furthermore, the EG presented increased breathing reserve (i.e., lower BRI) at the end of CPET. These results clearly show that CPET was able to identify subtle ventilatory changes despite the absence of ventilatory limitations at rest.

CPET, as previously described in the literature, has been used to assess ventilatory impairments and exercise tolerance in many types of pneumoconiosis, such as in mine workers (20), asbestos workers (21), and people with silicosis (22). However, those studies did not focus in early detection in asymptomatic subjects as our study. The most important result of the present study was the shallower ΔVT/ΔlnV̇E ratio in the EG, which suggests tachypneic breathing pattern accompanied with less ventilatory efficiency during exercise.

Sperandio et al. (23) observed worse ΔVT/ΔlnV̇E, lower peak V̇O_2_, V̇E, and VT in adolescents with idiopathic scoliosis with the incremental shuttle walk test when compared to control group. Reduction on the compliance of lung and/or chest wall cause changes in lung volumes and tachypnea during exercise (24). Furthermore, alterations on breathing pattern such as lower VT, increased respiratory effort, and rapid and shallow breathing were reported in individuals with interstitial lung diseases (25) and in patients with cystic fibrosis (26). Although the EG of the present study did not present evidence of scoliosis and spirometric restrictive pattern at rest, those participants exhibited a breathing pattern similar to that of patients with pulmonary restriction due to the limitation on the chest wall or intrapulmonary diseases. We believe that the difference in breathing pattern found between groups during CPET might be due to the exposure to a particulate matter. Thus, our results suggest that, among the several variables obtained in CPET, the ΔVT/ΔlnV̇E might be the most appropriate to differentiate workers with incipient ventilatory changes.

Changes in ΔVT/ΔlnV̇E might be a consequence of increased dead space to VT ratio (26,27). To attend the ventilatory demand during exercise, the ventilatory system uses combinations of respiratory rate and VT. At the initial phase, increasing ventilation occurs by increasing VT to close to 70–80% of inspiratory capacity. As exercise intensity progresses, ventilation continues to increase as respiratory rate increases. During exercise, there is also a similarity between ventilation and pulmonary perfusion, which reduces the volume of physiological dead space and, consequently, the ventilatory demand. In individuals with interstitial lung disease or reduction on the compliance of lung and/or chest wall, there is a decrease in pulmonary volumes and an increase in dead space, so the ventilatory strategy during exercise becomes less efficient, i.e., the increase in ventilation occurs predominantly due to the increase in respiratory rate (tachypneic pattern) (28). Thus, the worse ventilatory efficiency observed in workers with pneumoconiosis was justified by greater values of ventilatory equivalent of oxygen during exercise (i.e., V̇E/V̇O_2_), which might have resulted from increased V̇E (29). The shallower slope of ΔVT/ΔlnV̇E observed in the present study might be associated with lower VT, which leads to worse ventilatory efficiency in response to exercise.

Volpino et al. (30) evaluated pulmonary function and CPET performed on a cycle ergometer in workers exposed to urban pollution. Despite the different methodology from this study, the authors observed some similar results. In addition to reduced peak V̇O_2_ and AT, the exposed workers also showed significant worsening in ventilatory equivalent of CO_2_ (V̇E/V̇CO_2_) at AT and in the breathing reserve. These results indicate worse cardiorespiratory fitness associated with poor ventilatory efficiency in exposed participants. Although our study had a trend toward a higher proportion of participants with exercise intolerance, the results did not reach statistical significance. Volpino et al. (30) did not evaluate the breathing pattern as in the present study; we evaluated key submaximal relationships throughout CPET. The main advantage of the procedure proposed here is that submaximal relationships are not effort-dependent, i.e., even in low effort situations the early ventilatory changes described in the present study can be measured. Finally, ΔVT/ΔlnV̇E can be obtained using a simple ventilometry during ergometric tests, indicating the large potential of breathing pattern measurements to be routinely used for detecting early ventilatory abnormalities. This relationship clearly shows the breathing pattern during progressive exercise.

Differently from Volpino et al. (30), Schenker et al. (27) did not report a significant association between peak V̇O_2_ and OEPM. Inhalation of paraquat by harvest workers did not cause enough damage to lung function to result in exercise intolerance. However, they observed an association between the exposure to paraquat and worse V̇E/V̇CO_2_ at the end of the CPET performed on a cycle ergometer. These workers displayed oxygen desaturation during exercise, which is an indication of abnormalities in gas exchange, even in the absence of restrictive or interstitial lung impairments. Although peak V̇E/V̇CO_2_ is useful to identify ventilatory changes, the submaximal relations as presented in our study have the main advantage to perceive changes independent of maximum efforts, i.e., the ΔVT/ΔlnV̇E can be evaluated even in individuals with low cardiorespiratory fitness as well as in subjects with poor effort during the CEPT.

Spirometry has been used by many studies to evaluate pulmonary function in subjects with respiratory occupational exposure (4,31,32). However, the damages caused by OEPM do not always manifest in individuals at rest. Due to the increased metabolic demand under physical activity stress, impairments and alterations on the respiratory system become evident. Because altered spirometric indices are generally associated with alterations and disorders already installed, we believe that CPET is a useful tool for the early screening of ventilatory impairments in workers under OEPM.

Pulmonary function at rest was normal in both groups; nonetheless, CPET was able to identify discrete but significant changes in the breathing pattern during exercise. Discrepant results were reported by Bernardes et al. (33). Those authors found no difference in exposed and non-exposed lung function as measured by spirometry and functional capacity by the 6-minute walk test. According to the authors, the positive results were attributed to the use of personal protective equipment. We did not have information related to the use of appropriate protective equipment by our participants, which represent one of our limitations.

This study has some limitations. The EPIMOV study had the main purpose of diagnosing obstructive lung disease through the FEV_1_/FVC <70%. For this reason, we did not perform diffusion capacity measurements and measurements of total lung capacity at rest. We were unable to assess changes of the relationship between dead space volume and VT. Blood gases analyses were not carried out in our epidemiological study, which would have substantially enriched our results. Another limitation is the self-reported OEPM. The workers reported exposure to a large range of materials related to inhalable particles found in port activities including truck traffic and transportation of grains and cereals. However, we do not have precise information regarding the type of gases nor the time of exposure. Finally, even with the air quality annually classified as moderate (41–80 µg/m^3^) to good (0–40 µg/m^3^), the urban pollution could have affected the subjects as well, since the port area of Santos has a high concentration of MP10 (34).

According to Ramos et al. (35), the level of physical activity influences the inhaled doses of air pollution due to increased inhalation rates. The devices we used to track physical activity level of our participants were unable to specify physical activity of labor time with no information regarding a labor schedule. Therefore, we did not use physical activity compared to intensity of the labor and of the inhalation of particulate material. Moreover, we assessed the influence of moderate-to-vigorous physical activity as a continuous variable instead of a dichotomous variable for physical inactivity in the multivariate analysis, and found no differences in the results.

Despite these limitations, our results indicated excellent validity since after adjusting for confounder variables in the multivariate regression model, OEPM remained an independent predictor of the worst breathing pattern. Therefore, one simple question could track subjects with subclinical ventilatory changes. The results presented here should be interpreted with caution for women, given the gender differences in physiological responses to CPET (15).

We concluded that CPET is a promising tool for screening workers under OEPM, with the capacity to identify incipient ventilatory changes. CPET might be a more sensitive feature for assessing early pulmonary impairment related to OEPM, useful for the prevention of occupational respiratory diseases in asymptomatic workers.
